# Editorial: Epigenetics and stem cell therapy in cancer and diseases

**DOI:** 10.3389/fmmed.2025.1621093

**Published:** 2025-05-16

**Authors:** Sonia Coni, Roberto Schirano, Camilla Manfredi, Paola Infante, Veronica Veschi

**Affiliations:** Department of Molecular Medicine, University of Rome La Sapienza, Rome, Italy

**Keywords:** stem cells, stem cell therapy, cancer stem cells, epigenetics, cancer, diseases

## Introduction

Stem cell therapy and epigenetics of stem cells represent two deeply interconnected areas of biomedical research, especially in the context of cancer and diseases. Stem cell therapy involves pluripotent or multipotent stem cells to repair or replace damaged tissues. The most common sources include embryonic stem cells (ESCs), adult stem cells such as hematopoietic stem cells (HSCs), and induced pluripotent stem cells (iPSCs). In cancer research field, hematological cancers are commonly treated with bone marrow transplants using HSCs, mesenchymal stem cells (MSCs) are being engineered to deliver anti-cancer agents within the tumor bulk, iPSCs offer patient-specific cancer models or regenerative treatments post-therapy. In addition, stem cell therapy has shown advantages also in neurodegenerative diseases such as Parkinson’s and amyotrophic lateral sclerosis (ALS), or in cardiovascular diseases, autoimmune disorders and diabetes. In the field of regenerative medicine, the role of epigenetic regulation in maintaining stem cell pluripotency and directing lineage specification is a topic of active investigation. These processes are critical in human embryonic stem cells (hESCs), where dynamic chromatin remodeling governs both self-renewal and differentiation potential. Epigenetic dysregulation in stem cells is linked to developmental disorders, aging, and chronic inflammatory diseases.

In tumors, cancer stem cells (CSCs) which represent a particularly aggressive subpopulation characterized by self-renewal, resistance to therapy and metastatic capacity, are often regulated by aberrant epigenetic mechanisms. Uncovering epigenetic changes specific to CSCs is essential for identifying novel therapeutic strategies aimed at eradicating these resilient cells.

### Stem cell therapy as a promising option for regenerative medicine in cancer and diseases

Yang H et al. recognized MSCs as a promising cell therapy in acute liver failure (ALF), for the treatment of complications of liver transplantation, liver cancer, cirrhosis and liver failure caused by HBV, HCV, alcohol, primary biliary cholangitis and other infections. Yang H et al. dissect the potential benefits and limitations, future perspectives and challenges of MSCs use for the treatment of these diseases.

Yang J et al. discuss the potential of human amniotic epithelial cells (hAECs) non-invasively isolated from discarded placental amnion as a unique, promising source for regenerative medicine. Unlike ESCs, iPSCs and MSCs, which face hurdles in clinical translation due to tumorigenicity, immunogenicity, ethical debate, and invasive harvest procedures, hAECs are ethically uncontroversial, non-tumorigenic, exhibit low immunogenicity, with encouraging safety and efficacy data emerging from early-phase clinical trials. However, despite these advantages, hAECs pose significant challenges for large-scale application. Their inherently low telomerase activity limits proliferative capacity, and the absence of standardized markers and quality-control protocols impedes reproducible manufacturing. To overcome these barriers, a deeper understanding of hAEC biology, including the iPSC-like epigenetic landscape, and miRNA-mediated post-transcriptional regulation, is essential. As of January 2024, twenty-five clinical trials are evaluating hAECs’ paracrine effects, such as secretion of neurotrophic and growth factors, catecholamines, and dopamine, in neurological disorders (Parkinson’s and Alzheimer’s), immune diseases and tissue repair (NCT04414813; NCT05435755; NCT05691114; NCT03207412; NCT04728906; ACTRN12616000437460) (Műzes and Sipos, 2019). Preliminary data support their therapeutic potential in myocardial infarction, cerebral hemorrhage, retinal degeneration, and lung injury. To translate hAECs into a robust commercial product, concerted efforts must address scalable expansion, rigorous phenotypic characterization, and industrial-grade manufacturing processes. Such advances will bridge the gap between bench and bedside, fulfilling the promise of safe, effective, and ethically sound cell therapy, opening new scenarios in the field of stem cell therapy.

### Dissecting the role of epigenetic modifications and their contribution to diseases

Mengistu D.Y. et al. highlight how DNA methylation, histone modifications, and microRNAs (miRNAs) regulate the expression of the TARDBP gene, encoding TDP-43, an RNA-binding protein that is a pathological hallmark of ALS, and how the epigenetic deregulations contribute to disease progression. Both gain and loss-of-function of TDP-43 are implicated in ALS, yet the precise balance required for neuronal integrity remains unclear. DNA methylation has been shown to regulate TARDBP expression and isoforms. *In vitro*, knockdown of DNA methyltransferases (DNMTs) correlates with increased TDP-43 levels. In aging murine brains, increased repressive H3K27me3 and decreased H2A.Z acetylation at the TARDBP promoter reflect a more condensed chromatin structure and reduced gene expression. A conserved mechanism has been identified in *Drosophila*, where age-dependent H3K9me3 deposition at the TBPH promoter (TDP-43 ortholog) by the histone methyltransferase Su(var)3–9 suppresses gene expression and impairs locomotion. Genetic ablation of Su(var)3–9 reverses this phenotype, and similar effects are seen in human cells, suggesting a conserved regulatory axis. Finally, miRNAs contribution to this regulatory network was analyzed. Several miRNAs affect TARDBP expression, and ASL-related proteins are involved in miRNA biogenesis, suggesting a feedback mechanism.

These findings delineate an evolutionarily conserved epigenetic framework governing TDP-43 expression. Characterizing and manipulating this network may offer novel therapeutic strategies to restore TDP-43 homeostasis and modify disease progression in ALS.

### Epigenetic drugs targeting cancer stem cells to enhance the efficacy of conventional therapy

Recently, many epigenetic modifications have been involved in initiating and maintaining CSCs in various pediatric and adult cancer types (Veschi et al., 2019; Veschi and Thiele, 2017; Veschi et al., 2020; Veschi et al., 2025).

Verona F et al. overview the epigenetic modifications specifically present in CSCs, how these epigenetic alterations contribute to the maintenance of stemness and the initiation and progression of tumors, report on the most efficacious epigenetic probes and compounds that have been tested in clinical trials, either alone or in combination with standard or immune-based therapies. DNMT inhibitors like azacitidine and decitabine have shown promise in targeting CSCs and enhancing the efficacy of conventional treatments. In addition, DNMT1 and DNMT3 inhibitors, HDAC inhibitors, SIRT-1 agonists, and PRMT5 inhibitors have been used to target epigenetic aberrations specifically enriched in the CSC aggressive subpopulation in clinical or preclinical settings. Nwabo Kamdje et al. dissect the epigenetic modifications in hematological malignancies (HM), along with HM risk factors, treatment options, and prevention associated with the aberrant epigenetic mechanisms described.

## Conclusion

**FIGURE 1:**
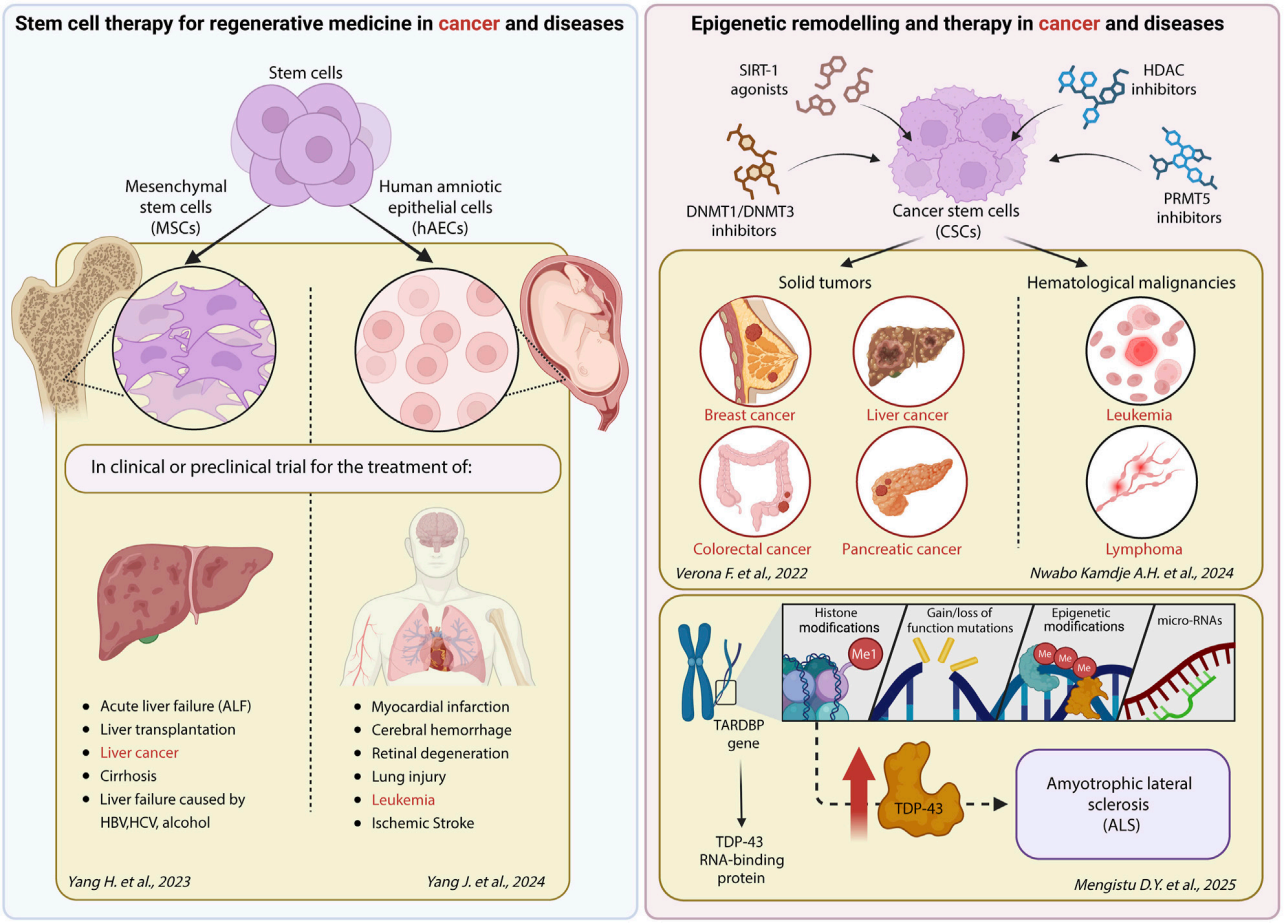
An overview of the main studies of this Research Topic. Created in BioRender. Giannini, G. (2025) https://BioRender.com/nbnw2ni.

Advancing our understanding about the altered epigenetic mechanisms and the compounds targeting them that have progressed to clinical application could improve standard therapies, enabling the development of bioengineered stem cell-based regenerative interventions in cancer and diseases (Figure 1).
